# Characterization of the Flash-Induced Fluorescence Wave Phenomenon in the Coral Endosymbiont Algae, Symbiodiniaceae

**DOI:** 10.3390/ijms24108712

**Published:** 2023-05-13

**Authors:** Sabit Mohammad Aslam, Imre Vass, Milán Szabó

**Affiliations:** 1 Institute of Plant Biology, Biological Research Centre, Eötvös Loránd Research Network, 6726 Szeged, Hungary; 2 Doctoral School of Biology, Faculty of Science and Informatics, University of Szeged, 6720 Szeged, Hungary; 3 Climate Change Cluster, University of Technology Sydney, Ultimo 2007, Australia

**Keywords:** photosynthesis, chlorophyll fluorescence, symbiotic microalgae, linear electron flow, cyclic electron flow

## Abstract

The dinoflagellate algae, Symbiodiniaceae, are significant symbiotic partners of corals due to their photosynthetic capacity. The photosynthetic processes of the microalgae consist of linear electron transport, which provides the energetic balance of ATP and NADPH production for CO_2_ fixation, and alternative electron transport pathways, including cyclic electron flow, which ensures the elevated ATP requirements under stress conditions. Flash-induced chlorophyll fluorescence relaxation is a non-invasive tool to assess the various electron transport pathways. A special case of fluorescence relaxation, the so-called wave phenomenon, was found to be associated with the activity of NAD(P)H dehydrogenase (NDH) in microalgae. We showed previously that the wave phenomenon existed in Symbiodiniaceae under acute heat stress and microaerobic conditions, however, the electron transport processes related to the wave phenomenon remained unknown. In this work, using various inhibitors, we show that (i) the linear electron transport has a crucial role in the formation of the wave, (ii) the inhibition of the donor side of Photosystem II did not induce the wave, whereas inhibition of the Calvin–Benson cycle accelerated it, (iii) the wave phenomenon was related to the operation of type II NDH (NDH-2). We therefore propose that the wave phenomenon is an important marker of the regulation of electron transport in Symbiodiniaceae.

## 1. Introduction

Symbiodiniaceae is a family of unicellular microalgae (Dinophyceae) that mostly live in a mutualistic arrangement with cnidarian hosts, such as anemones and jellyfish [1], contributing to the primary productivity of the entire reef ecosystem. Since reefs are mostly found in low-nutrient-content seawater [2], these microalgae play a crucial role in supporting the energy needs of the host via photosynthesis, while the host absorbs and supplies nutrients to them. This mutualistic relationship is the fundamental basis of the functionality of the entire reef ecosystem [3].

The light-harvesting antenna complex of Symbiodiniaceae mainly comprises chlorophyll a, chlorophyll c2, and carotenoids such as peridinin (Per) and diadinoxanthin (Ddx) [4]. The process of photosynthesis remains conserved from unicellular organisms to higher plants, where light energy is converted to chemical energy upon the expenditure of CO₂ and water. The light energy is absorbed by the light-harvesting antenna system, and then transferred to the reaction centers of Photosystem II and I (PSII and PSI, respectively), where charge separation occurs. The electrons from excited PSII reduce plastoquinone with the help of pheophytin, and further, the reduced plastoquinones transfer it to the cytochrome b_6_/f (Cytb₆f) complex, which provides a connection between PSII and PSI. In PSI, the light-induced charge separation reduces the 4Fe-4S cluster, from which the electrons are passed to Ferredoxin (Fd) and finally to Ferredoxin-NADP^+^ Reductase (FNR), which in turn reduces the NADP to NADPH. The oxidized reaction center of PSII is consequently re-reduced by the oxygen-evolving complex (OEC), while that of PSI is re-reduced by the Cytb₆f complex via plastocyanin (or cyt c_6_ in dinoflagellates) [5]. Meanwhile, the accumulated protons in the lumen from OEC and Cytb₆f generate a proton motive force (pmf) that drives the synthesis of ATP molecules. These NADPH and ATP molecules act as energy carriers for the Calvin–Benson (CB) cycle, where type II RubisCo performs the final carbon fixation [6].

In general, two molecules of NADPH and three ATP molecules are required to complete one CB cycle. Given that the mechanism of photosynthesis remains conserved, 14 H^+^ ions are needed to produce 3 ATP molecules [7,8]. Since only 12 H⁺ ions (4 from OEC and 8 from Cytb₆f) can be generated from the linear electron transport pathway from water to NADPH, alternative electron transport mechanisms are required to adjust the production of NADPH and ATP under normal, as well as stress, conditions. The main alternative pathways are cyclic electron flow (CEF) and the water-to-water cycle (WWC), also known as pseudo-cyclic electron flow [9].

CEF consists of PSI, plastoquinone (PQ), and the Cytb₆f complex. Electrons that are generated from the donor side of the excited PSI, instead of going to FNR, are transferred to the PQ pool. They further follow the linear pathway, where the electrons are transferred to Cytb₆f, which in turn returns them to the donor side of PSI with the help of mobile carrier PC (or cyt c_6_). In this process, no net NADPH is formed, but it increases the H^+^ concentration in the lumen, hence providing more driving force for ATP production. Depending upon the route of the electrons, CEF can be divided into four different pathways. These are mediated by (i) the NADPH dehydrogenase complex 1 (NDH1) [10], (ii) type II NADPH dehydrogenase (NDA2) [11], (iii) Protein Gradient Regulator-Like 1 (PGRL1)/ Protein Gradient Regulator 5 (PGR5) [12], and (iv) direct transfer from Fd to the cytochrome b₆f complex [13]. The genes coding for PGR5 and PGRL-1 (FQR) and for NDH2 have been found in Symbiodiniaceae [14,15], but the actual processes behind them are still uncharacterized.

On the other hand, the water-to-water cycle (WWC) comprises both PSII and PSI, plastoquinone (PQ), and the cytochrome b_6_/f complex. Electrons at the acceptor side of P700 can participate in reducing the oxygen molecules generated from splitting water in the OEC back to water. In Symbiodiniaceae, there are two known pathways: one involves FLV protein, which uses NADPH to reduce the oxygen molecules [16,17]; the other pathway is called the Mehler–Asada pathway, where enzymes such as superoxide dismutase (SOD) and ascorbate peroxidase (APX) help in producing H₂O, with ascorbate as the reductant, and the enzyme mono-dehydroascorbate reductase (MDAR) uses NADPH to restore the ascorbate back to the cycle [18]. Hence, this process also helps in increasing the pmf, but upon the expenditure of NADPH.

A phenomenon called coral bleaching, where coral hosts expel the microalgae present in their membrane, has been widely observed across the globe in the last few decades. Effects of various biotic and abiotic stresses leading to coral bleaching have already been well documented in the literature. One such stress is the increase in the global temperature. As per the ncdc-Global Climate Report—Annual 2020 [19], the rate of increase in the global temperature has more than doubled when comparing the past decades. Research has shown that increased temperature has an adverse effect on the Symbiodiniaceae. It involves a range of physiological and biochemical impacts that also affect the photosynthetic apparatus, damage Photosystem II, inactivate the Calvin–Benson cycle, etc., whereas other components, such as Photosystem I, were found less sensitive to the damage [20].

Alternative electron flow processes, such as WWC and CEF, have been shown to operate in Symbiodiniaceae as well, however, their photo-protective capacity differs among the different species, and if their photo-protective capacity is exceeded by the level of stress, photo-oxidative damage to the photosynthetic processes might occur [21]. The intersystem PSII–PSI electron transfer processes, the alternative electron flow, and the interconnection of the photosynthetic electron transport with other physiological processes can be readily studied with a combination of methods, such as variable chlorophyll fluorescence, kinetic absorption spectroscopy, and gas exchange [15,20,22,23].

Flash-induced chlorophyll fluorescence relaxation kinetics is a powerful technique to study the electron transport around PSII. This technique involves measuring flashes, and a short single-turnover actinic flash causing the transient formation of Q_A_^−^, which in turn increases the fluorescence yield [24,25,26]. Further, the decrease in fluorescence is monitored over a time range of microseconds to seconds. This decay in fluorescence is divided into three different phases, including the fast phase in the first 300–500 µs, which is dominated by the electron transfer to Q_B_, the middle phase of around 5–15 ms, reflecting the reoxidation by the plastoquinone that binds to the Q_B_ site from the PQ pool, and the slow phase of around 10–20 s, which reflects the reoxidation of Q_A_^−^ via charge recombination with the oxidized S₂ (or S₃) states of the donor side of PSII [25,27]. A special case of a wave-like decay pattern, which is formed by the re-reduction and oxidation of the PQ pool in the microaerobic condition, is well characterized in *Synechocystis* sp. [27,28]. However, this phenomenon was found to be related the ratio of PSII: PSI, as microaerobic condition alone was not sufficient to induce the wave in eukaryotic microalgae [29,30,31,32,33,34], and the intensity of the wave was also found to be species-specific within the family Symbiodiniaceae [34]. The exact details of electron transfer processes that give rise to the wave phenomenon, i.e., the operation of linear electron flow, the redox reactions of the PQ pool, and the involvement of alternative electron transfer processes (such as CEF), have not been clarified so far in Symbiodiniaceae. The combination of flash-induced fluorescence relaxation with P700 redox kinetics, which provides information about the electron transport through and around PSI, becomes a crucial approach to investigate the whole-chain electron transport processes. Furthermore, by using specific chemical inhibitors of electron transfer, the involvement of various sections of linear and cyclic electron flow, as well as the involvement of the CB cycle, could be revealed.

The aim of the current work was to investigate the flash-induced Chl fluorescence relaxation phenomenon in Symbiodiniaceae by using inhibitors that are specific to certain electron transport processes. We aimed to investigate (i) the involvement of the linear electron flow, (ii) the impacts of the inhibition of the donor side of PSII and the Calvin–Benson cycle, and (iii) the contribution of the PGR5/PGRL1 and NDH-2 pathways to the wave phenomenon. The specific chlorophyll fluorescence signals in combination with P700 redox kinetics provide useful insights into the significance of these largely unknown pathways in Symbiodiniaceae.

## 2. Results

### 2.1. Effect of Blocking Linear Electron Flow

First, it was investigated whether the blockage of the linear electron transport by DCMU influences the wave (induced by acute heat + microaerobic treatment, [34]).

**Figure 1:**
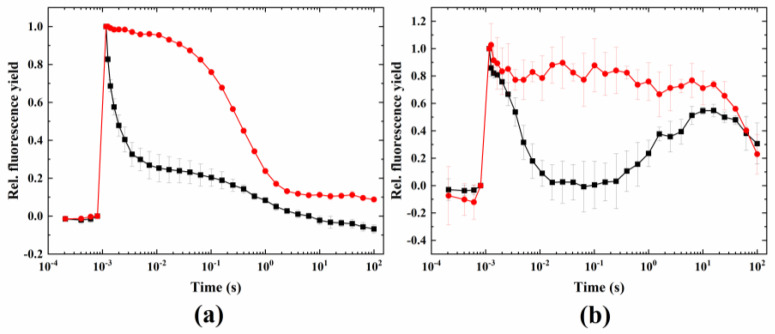
Effect of DCMU on the wave phenomenon of flash-induced Chl fluorescence relaxation. Treatments were conducted under (**a**) 24 °C (black) plus DCMU (red) and (**b**) 38 °C + microaerobic (black) plus DCMU (red) conditions.

Under ambient temperature, DCMU completely blocked the fast and middle phase of fluorescence relaxation after the flash; only the slow phase remained (Figure 1). Under the conditions that induce the wave phenomenon (heat + microaerobic treatment), the wave, i.e., the dip in fluorescence relaxation in the couple of hundreds of milliseconds after the flash followed by a decline in the fluorescence yield, was absent (although a small decrease in the fast phase in 38 °C + microaerobic treatment remained). This is because the electron transfer between Q_A_ and Q_B_ was blocked by DCMU, and therefore, one of the essential conditions of the formation of the wave, namely, the undisturbed operation of sequential electron transfer between PSII and the PQ pool [27], was not fulfilled.

**Figure 2:**
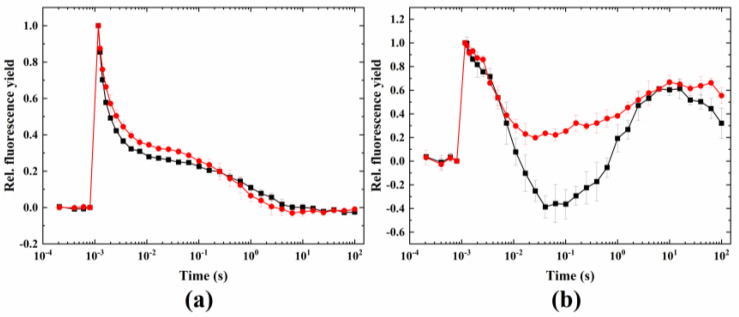
Effect of DMBQ on the wave phenomenon of flash-induced Chl fluorescence relaxation. Measurements were taken under (**a**) 24 °C (black) plus DMBQ (red) and (**b**) 38 °C + microaerobic (black) plus DMBQ (red) conditions.

DMBQ, a soluble electron acceptor to PSII that keeps the PQ largely oxidized, caused minor changes in the fluorescence relaxation under control conditions (Figure 2). Under wave-inducing conditions (38 °C + microaerobic), the dip below the original F_0_ level was blocked, however, the wave phenomenon did not fully disappear (unlike in *Synechocystis*, where a complete elimination of the wave was observed with DMBQ treatment).

**Figure 3:**
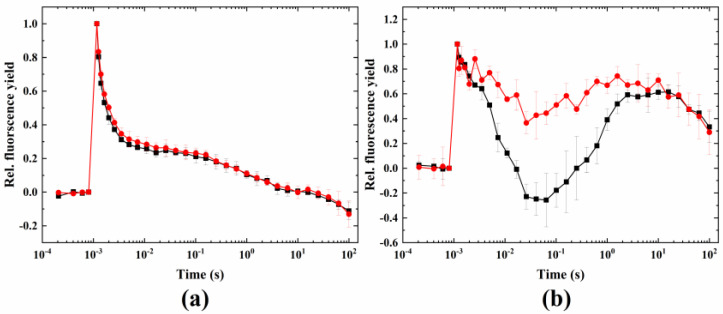
Effect of methyl-viologen on the wave phenomenon of flash-induced Chl fluorescence relaxation. Measurements were taken under (**a**) 24 °C (black) plus methyl-viologen (red) and (**b**) 38 °C + microaerobic (black) plus methyl-viologen (red) conditions.

Methyl-viologen, an electron acceptor that competes with ferredoxin for electrons from the FeS clusters at the acceptor side of PSI, and therefore suppresses CEF [35], did not have any effect of the fluorescence relaxation under normal conditions. Under wave-inducing conditions, it largely blocked the wave phenomenon, and only a small dip could be observed, however, the fluorescence level did not drop below the F_0_ level (Figure 3).

### 2.2. Effect of Blocking the Primary Electron Donor of PSII and the Terminal Electron Acceptor in CO_2_ Fixation

In order to test a condition where the PSII activity relative to the PSI activity was decreased, a hydroxylamine (HA) treatment was applied at ambient temperature (24 °C), either under aerobic or under microaerobic conditions.

**Figure 4:**
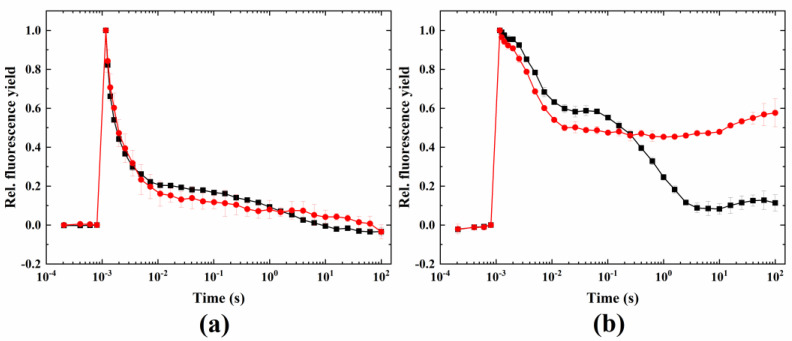
Effect of hydroxylamine on the flash-induced Chl fluorescence relaxation profile. Treatment was conducted under (**a**) 24 °C (black) plus hydroxylamine (red) and (**b**) 24 °C + microaerobic (black) plus hydroxylamine (red) conditions.

HA did not have a pronounced effect on the fluorescence relaxation after the flash as compared to the non-treated control (Figure 4). Under microaerobic conditions and HA treatment, a strongly reduced PQ pool was observed (increased middle phase). However, unlike in *Chlamydomonas* [32], the large dip below F_0_ could not be observed, and only a small increase in the Chl fluorescence (in the 1–100 s timescale) occurred. In HA-treated samples, the wave phenomenon characteristic to the heat + microaerobic treatment was absent (the lack of the slow component of the relaxation in the seconds timescale is due to the lack of a functional donor side of PSII after the HA treatment). The fluorescence relaxation profile under HA + microaerobic treatment at 24 °C is similar to the curves obtained upon heat treatment, which also caused damage to the donor side of PSII [34].

Under conditions where the terminal electron acceptor for CO_2_ fixation is blocked, and therefore, linear electron flow from PSI towards the CB cycle is inhibited, but the alternative electron flow processes, such as CEF, operate unimpeded or even accelerated, the wave phenomenon is expected to display faster phases. This was tested by inhibiting the CB cycle using glycolaldehyde (GA).

**Figure 5:**
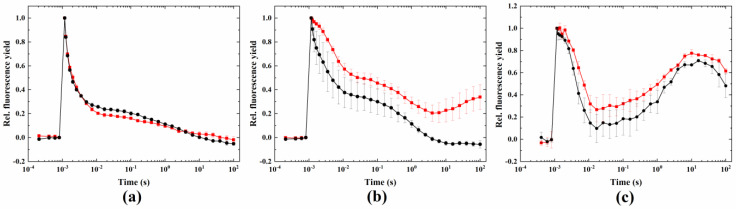
Effect of glycolaldehyde (GA) on the flash-induced Chl fluorescence relaxation profile. Treatment was conducted under (**a**) 24 °C (black) plus glycolaldehyde (red), (**b**) 24 °C + microaerobic (black) plus glycolaldehyde (red), and (**c**) 38 °C + microaerobic (black) plus glycolaldehyde (red) conditions.

GA did not cause a significant change in the fluorescence relaxation profile at 24 °C aerobic condition (Figure 5a). Furthermore, GA did not cause significant changes in the fluorescence wave that was induced under heat + microaerobic conditions (Figure 5c). A possible reason for this is that, under these conditions, the wave phenomenon, i.e., the transient reduction–oxidation and re-reduction of the PQ pool operates at its maximal rate, therefore, blocking the CB cycle could not accelerate it further (and possibly, the CB cycle was also inhibited by the heat treatment itself).

However, when GA was added at ambient temperature (24 °C) to microaerobic samples, a wave-like phenomenon was observed, with a dip at around 4 s and a subsequent increase in fluorescence (Figure 5b). This dip was absent in microaerobic but non-GA-treated cells (at 24 °C), even though the PQ pool was strongly reduced due to the microaerobic treatment [34].

In order to investigate the effect of glycolaldehyde on the P700^+^ re-reduction kinetics, P700 absorption measurements were carried out in the presence of DCMU, when the electron flow from PSII towards PSI was blocked, and therefore, PSI-CEF dominated [15,36].

**Figure 6:**
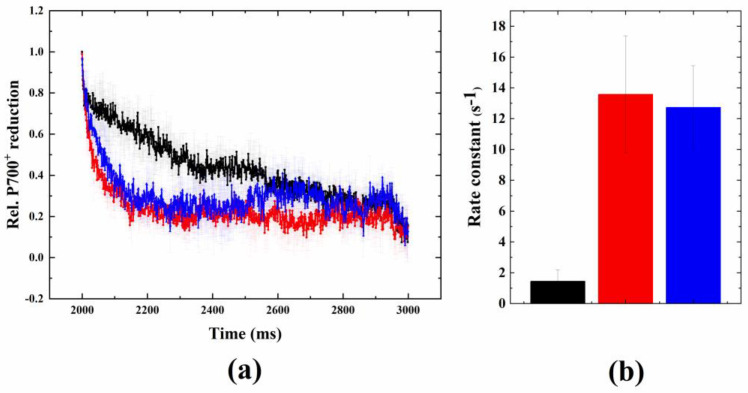
Effect of glycolaldehyde or heat treatment on the re-reduction kinetics of P700^+^. PSII activity was blocked by DCMU in order to investigate cyclic electron flow around PSI. (**a**) Original traces (mean ± S.D.), including control, i.e., 24 °C + DCMU (black), 38 °C + DCMU (red), and control + glycolaldehyde (blue), and (**b**) respective rate constants of P700^+^ re-reduction (from exponential fitting).

As compared to the non-treated control cells, glycolaldehyde significantly induced the acceleration of P700^+^ re-reduction in DCMU-poisoned cells at ambient temperature. This acceleration could also be observed with acute heat treatment to a similar extent (Figure 6a). In both glycolaldehyde- and acute-heat-treated cells, the rate of P700^+^ re-reduction increased 8–10 fold as compared to untreated controls (Figure 6b).

### 2.3. Effect of Cyclic Electron Flow Inhibitors

**Figure 7:**
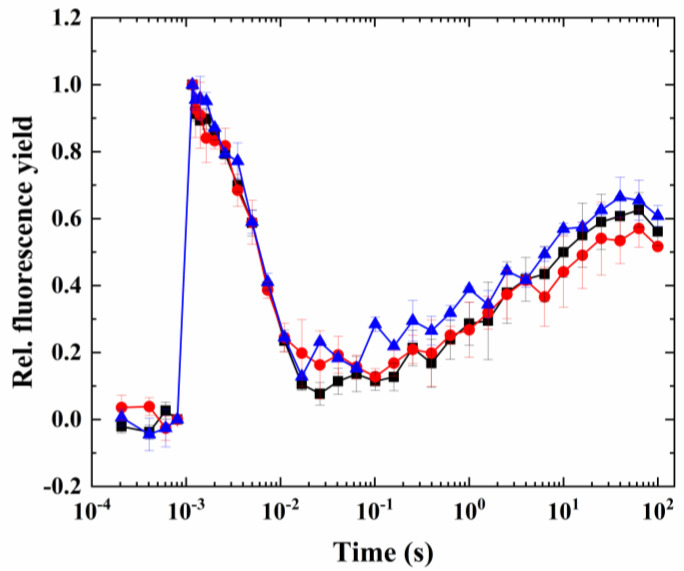
Effect of antimycin A and polymyxin B on the flash-induced Chl fluorescence relaxation profile. Treatment is indicated as control, i.e., 38 °C + microaerobic (black), control + polymyxin B (red), and control + antimycin A (blue).

Antimycin A (inhibitor of the PGR5/PGRL1 pathway) and polymyxin B (inhibitor of the NDH-2 pathway) did not have any significant effect on the wave phenomenon in Symbiodiniaceae (Figure 7). These inhibitors did not considerably affect the P700^+^ re-reduction kinetics either (Supplementary Figure S1). However, it is possible that these inhibitors do not penetrate intact *Symbiodinium* cells due to the presence of a thick cell wall, as was previously suggested for antimycin A [15].

Therefore, in order to test these inhibitors in the absence of the cell wall, protoplasts were prepared by digestion of the cell wall using cellulase, applying the modified protocol of Bashir et al. 2022 [37]. As the protoplast preparation is a considerable stress to the cells, the cell wall digestion procedure that was applied previously (i.e., a complete cell wall digestion with 4% cellulose) significantly affected the fluorescence relaxation, and the wave disappeared under these conditions (Supplementary Figure S2). Therefore, the cell wall digestion procedure had to be optimized to preserve photosynthetic activity and to retain the typical fluorescence wave feature of *Symbiodinium*. Partial digestion with 2% cellulase (Section 4.2) retained the characteristic features of the flash-induced fluorescence relaxation and the wave phenomenon, and therefore, allowed us to investigate the effect of CEF inhibitors.

**Figure 8:**
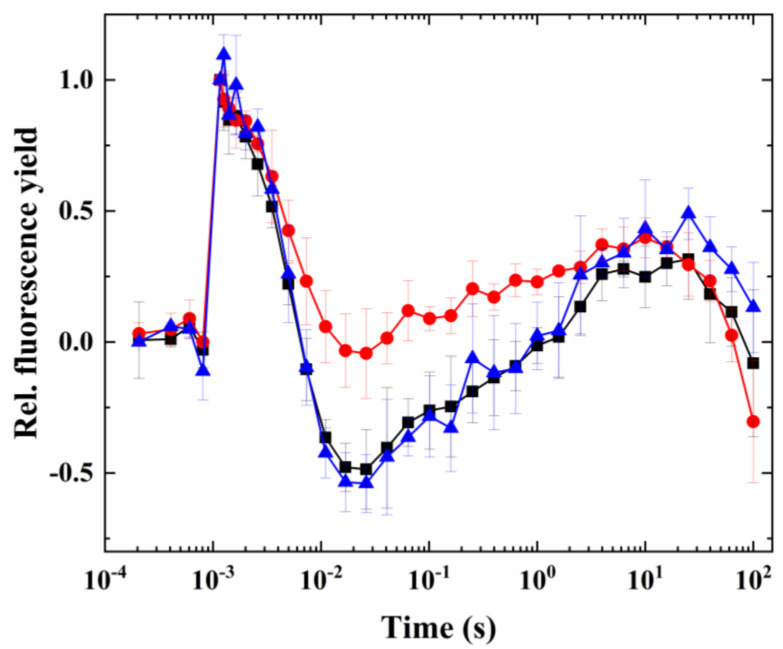
Effect of antimycin A and polymyxin B on the flash-induced Chl fluorescence relaxation profile in cells with partially digested cell walls. Treatment is indicated as control, i.e., 38 °C + microaerobic (black), control + polymyxin B (red), and control + antimycin A (blue).

**Figure 9:**
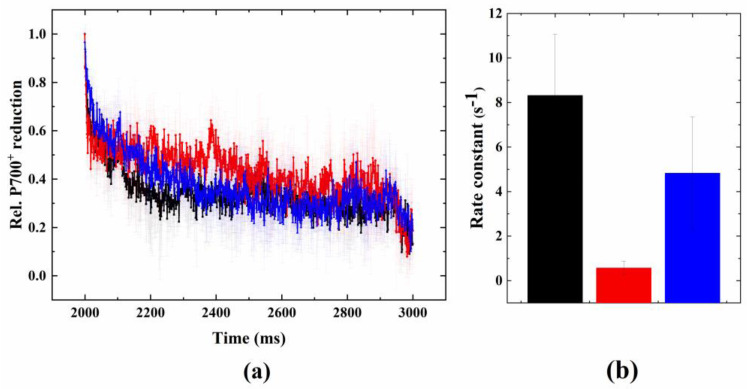
Effect on antimycin A and polymyxin B on P700^+^ re-reduction kinetics in cells with partially digested cell walls. Treatment was conducted at 38 °C in the presence of DCMU. (**a**) Original traces (mean ± S.D.), including control, i.e., 38 °C + DCMU (black), control + polymyxin B (red), and control + antimycin A (blue), and (**b**) respective rate constants of P700^+^ re-reduction (from exponential fitting).

Partially digested cells retained the characteristic fluorescence wave phenomenon (which was induced in the same way as in the case of intact cells, heat + microaerobic treatment; Figure 8, black trace). When the inhibitor treatments were conducted on partially digested cells, it was found that antimycin A did not have any inhibitory effect on the wave phenomenon, whereas polymyxin B inhibited the wave significantly (although it did not completely eliminate the fluorescence dip phase after the flash) (Figure 8). The larger inhibitory effect of polymyxin B could also be seen in P700^+^ re-reduction kinetics (Figure 9a and the calculated rate of P700^+^ re-reduction, Figure 9b), which indicates that the NDH-2 pathway may have a dominant role in electron transport between the acceptor side of PSI and the PQ pool via cyclic electron flow. It also has to be noted that the P700^+^ re-reduction kinetics were temperature-dependent in this species, in agreement with previous findings (Supplementary Figure S3).

## 3. Discussion

### 3.1. Linear Electron Flow Inhibitors Largely Blocked the Fluorescence Wave Phenomenon in Symbiodiniaceae

Flash-induced fluorescence relaxation has been shown to exhibit characteristic phases of the electron transfer processes. In addition to the typical three components of relaxation, the specific wave phenomenon in cyanobacteria also provides information about the operation of NDH-1 complexes [27]. As the wave phenomenon is far less characterized in microalgae, especially in the coral endosymbiont algae Symbiodiniaceae, it is essential to understand the behavior and relation of the components of fluorescence relaxation to various sections and processes of the photosynthetic electron transport chain; thereby, the flash-induced fluorescence relaxation method is a potentially informative and versatile tool to reveal specific patterns in the linear or alternative electron flow processes [27,28,31,38]. Since fluorescence relaxation kinetics contain information not only about the operation of PSII, but also about the operation of PSI and alternative electron sources that feed the entire electron transfer chain and the intersystem PSII–PSI electron transport network, this tool may reveal key regulatory checkpoints under stress conditions.

However, the appearance, characteristics, and the timescale of fluorescence relaxation exhibit important differences and variability between the different algal taxonomic groups [33]. Therefore, by revealing the flash-induced fluorescence patterns in algal species that have not been characterized in this aspect requires understanding not only the conditions that induce the wave pattern, but also the dissection of the involved electron transfer components. Since in Symbiodiniaceae, only one report exists on the induction of the wave phenomenon [34], it remained uncertain as to whether the wave phenomenon was related to linear or alternative (cyclic) electron flow. Therefore, selective inhibitors were applied in this study to tackle these questions.

As it has been shown earlier, one of the main conditions to induce the wave phenomenon is the functional linear electron transport from PSII to stromal electron carriers [27]. Similar to *Synechocystis*, DCMU eliminated the fast and middle phases and the relaxation pattern in Symbiodiniaceae, resulting in a typical pattern, in which only the slow phase (related to charge recombination between Q_A_^−^ and the S_2_ state of the water oxidizing complex) [25] could be observed (Figure 1a). As DCMU blocks the binding of PQ at the Q_B_ site of PSII, the fluorescence dip and subsequent rise was prevented, indicating that operational linear electron transport is essential in the formation of the wave phenomenon in Symbiodiniaceae as well.

Another inhibitor used for this aim was DMBQ, which intercepts electrons at PSII and prevents their delivery to PSI via the Cytb_6_/f complex (and so inhibits linear electron flow and keeps the PQ pool largely oxidized). DMBQ largely eliminated the dip in the fluorescence wave. This shows that inhibiting the linear electron flow by the inhibitors DCMU and DMBQ largely blocked the wave phenomenon, although a complete inhibition could not be observed in the case of Symbiodiniaceae. This could be due to the acute heat damage of the quinone-binding site of PSII, therefore, the effect of DCMU and DMBQ was not full, allowing the fluorescence wave to be partially retained. The fact that MV also eliminated the wave indicates the importance of the intact electron transport chain in the whole process (up to the CO_2_ fixation step). However, as MV intercepts electrons at the PSI acceptor side, the effect of this inhibitor manifests itself in the inhibition of CEF rather than LEF, because the electron withdrawal at the PSI acceptor side prevents the cycling of electrons from Fd (or NADH) back to the PQ pool.

### 3.2. Inhibiting the Donor Side of PSII and the Calvin–Benson Cycle Distinctly Affected the Wave Phenomenon

Inactivation of the donor side of PSII using hydroxylamine in combination with microaerobic treatment did not induce the wave phenomenon, unlike in the case of *Chlamydomonas reinhardtii* and other green algae, where a remarkable wave was induced under these conditions [32,33]. Inhibition of the donor side of PSII by hydroxylamine decreases PSII activity relative to PSI activity, which in combination with a strongly reduced PQ pool (microaerobic condition) causes a strong imbalance between the electron flow from PSII to the PQ pool and the electron withdrawal from the PQ pool towards PSI, eliciting the wave phenomenon in certain microalgae [32,33]. However, in Symbiodiniaceae, the wave phenomenon did not manifest itself under this condition, as compared to the acute heat + microaerobic treatment [34]. It has been shown earlier that upon elimination of the oxygen-evolving complex of PSII by sulfur deprivation (which induced H_2_-producing conditions) a characteristic wave phenomenon in *C. reinhardtii* was induced [31], showing the importance of reduced PSII activity relative to the PSI activity in the induction of the wave in this species. This was mimicked with HA treatment in *C. reinhardtii*, which also led to the appearance of the wave [32]. However, it appears that, in Symbiodiniaceae, the decrease in PSII activity relative to PSI activity was not sufficient to induce a similar wave phenomenon, even under microaerobic conditions, which could be due to an insufficient electron flow back to the PQ pool after the flash.

Therefore, the question arises, could the wave phenomenon be enhanced by accelerating the rerouting of the electrons from the acceptor side of PSI to the PQ pool by blocking the Calvin–Benson cycle? It was shown earlier that glycolaldehyde, the chemical inhibitor of the CB cycle, also functional in corals [39,40], slowed down the linear electron flow and caused elevated levels of NADPH and ferredoxin (due to the inhibited NADPH uptake of the CB cycle), which caused there to be an over-reduced PQ pool via cyclic electron flow and blockage of the PSI acceptor side [23]. GA under ambient conditions did not influence the fluorescence relaxation in our current study, possibly because the decrease in PSII activity relative to PSI activity was not sufficient enough to create an imbalance to the advance of the electron withdrawal from the PQ pool towards PSI. However, GA treatment induced a wave-like phenomenon under microaerobic conditions (at ambient temperature), indicating the importance of the highly reduced PQ pool. This was accompanied by a significant acceleration in P700^+^ re-reduction in the presence of GA, even at ambient temperature (in the presence of DCMU), indicating that the induction of the fluorescence wave is related to the activation of cyclic electron flow, in agreement with previous results (although some studies found that the effect of GA on the acceleration of P700^+^ re-reduction was only moderate [15,36]). The different experimental approaches of inducing a wave or wave-like phenomenon enabled us to better understand the characteristics of the fluorescence wave in Symbiodiniaceae. Our findings bring about the conclusion that it is not the elimination of the donor side activity that mainly determines the appearance of the wave phenomenon, but rather the blockage of the CB cycle and the concomitant impacts of PSI activity that induce a rise in this phenomenon in Symbiodiniaceae. This is supported by the finding that the impact of GA is similar to that of acute heat, as it leads to enhanced P700^+^ re-reduction (Figure 6).

However, it is also important to note that none of these experimental treatments induced the wave to its maximal extent, i.e., the case of the application of acute heat stress + microaerobic treatment. Therefore, it appears that in Symbiodiniaceae, the overall impacts of acute heat on both the donor of PSII and the acceptor of PSI (the CB cycle) is required for the full manifestation of the wave phenomenon. Although the acute heat stress temperature applied here is well above the typical seawater temperatures for growth of *S. tridacnidorum* and that induce coral bleaching (in the 32–33 °C range), under bleaching conditions, the corals are exposed to bleaching temperatures for a much longer time period, whereas in our experiments, the higher 38 °C treatment is applied for a short time. While exposure to the 38 °C treatment for a short time cannot really be considered ecologically relevant, it still gives important information about the condition when PSII activity decreases relative to PSI activity [34], the wave-inducing condition can be achieved under this temperature (i.e., 38 °C or higher). Nonetheless, these conditions can be considered as environmentally relevant in certain cases, because corals may experience extreme heat events up to 38 °C (as reviewed in [41]). High heat stress may also result in the establishment of anoxic conditions, due to the elevated respiratory activity of both the host and the algal symbiont, and several other factors operating in isolation or combination [42].

### 3.3. The Role of NDH-2-Mediated Electron Flow in the Wave Phenomenon

In order to investigate which components of cyclic electron flow might be participating in the induction of the wave phenomenon, inhibitors of the antimycin-sensitive (PGR5) and -insensitive (NDH-2) pathways (antimycin A and polymyxin B, respectively) were applied. However, even though the coding genes of both pathways were found in Symbiodiniaceae, antimycin A proved inefficient in blocking the cyclic electron flow or NPQ [15]. This is in agreement with our findings that antimycin A was inefficient in blocking the wave and the P700^+^ re-reduction kinetics. This was the case for the NDH-2 inhibitor polymyxin B as well. However, the cell wall might be impermeable for these inhibitors, therefore, their effect cannot be judged in intact cells. To avoid the hinderance of the cell wall in the uptake of antimycin A and polymyxin B, the cell wall was removed using enzymatic digestion with cellulase [37,43]. In cells with a partially digested cell wall, polymyxin B blocked the wave phenomenon and slowed down the P700^+^ re-reduction kinetics, whereas the inhibitor of PGR5, antimycin A, did not have considerable effect. Therefore, it appears that in the case of Symbiodiniaceae, NDH-2 contributes to the formation of the wave phenomenon by mediating the electron transfer between NADPH and the PQ pool, similar to *C. reinhardtii* and some other green algae [31,32].

As it has been analyzed and discussed earlier [34], the relatively slow increase in fluorescence after the dip, occurring in the seconds timescale, is not commensurable with the rate of CEF; therefore, it is plausible to assume that the wave phenomenon cannot be directly assigned to the operation of CEF. It was also found that the rate of PGR5 and NDH-2 is too slow to assign the operation of these enzymes to CEF [13,44]. However, the extent and timescale of CEF can be varied over a broad range [13,44]; therefore, it is possible that there are different CEF scales, which may be modulated by the NDH-2 pathway. Furthermore, it is likely that electron donation from Cytb_6_/f and PSI operates at the rate that is typical for CEF (indicated by the P700^+^ re-reduction in the 50–100 ms timescale under heat + microaerobic conditions). However, the electrons involved in the re-reduction of the PQ pool—which is in a much longer timescale, indicated by the increase in the fluorescence after the dip, in the seconds timescale—originate from alternative sources from the stroma rather than mediated by the CEF. This slow PQ pool re-reduction, however, could be readily mediated by the NDH-2 pathway, as indicated by the inhibitory effect of polymyxin B. Based on the characteristics of the fluorescence wave phenomenon as investigated in the current study (and supported by the P700^+^ re-reduction kinetics results), it appears that different electron sources with vastly different kinetics play a role in the overall formation of the wave, and NDH-2 has a more direct regulatory function on the slow alternative electron transport than the PGR5/PGRL1 pathway in Symbiodiniaceae. However, it cannot be excluded that both systems have important functions under certain conditions; for a full understanding of the regulation of the CEF and other alternative electron transport processes in Symbiodiniaceae, the role of the NDH-2 and the PGR5/PGRL1 system has to be systematically investigated in the future, under environmentally relevant, complex stress scenarios.

In conclusion, in the current work, we demonstrate, by using specific inhibitors, that the wave phenomenon of flash-induced Chl fluorescence relaxation critically depends on the operational linear electron flow in Symbiodiniaceae. The wave is accelerated when the Calvin–Benson cycle is inhibited along with accelerated P700^+^ re-reduction, indicating the enhancement of cyclic electron flow when CO_2_ fixation is blocked. The wave phenomenon was also found to be dependent on a slow, stromal-electron-source-related alternative electron transport, mediated by the operation of NDH-2 but not by the PGR5/PGRL1 components. Therefore, this work clarifies, for the first time, the involvement of several electron transport pathways in the formation of the fluorescence wave phenomenon, which, by this means, could be an important non-invasive marker of photosynthesis remodeling in coral endosymbiont algae under various environmental conditions. 

## 4. Materials and Methods

### 4.1. Symbiodiniaceae Cultures

*Symbiodinium tridacnidorum* (CCMP2465), formerly clade A3, was grown under white light with intensity of 50 µmol photons m^−2^ s^−1^ for 1 week at 24 °C in F/2 media until reaching the mid-log growth phase. Cells were centrifuged at 5000× *g* and re-suspended in fresh F/2 media. The final chlorophyll (Chl) concentration was calculated on the basis of extraction with 100% methanol [45], was adjusted to 5 µg/mL for flash-induced chlorophyll fluorescence, and 20 µg/mL for P700^+^ reduction kinetics measurements. Prepared samples were maintained under growth light and temperature conditions for 1 h to acclimate before each measurement.

### 4.2. Partial Digestion of Symbiodinium Tridacnidorum

Protoplasts were prepared using a modified protocol according to [37] (sorbitol was avoided and the cellulase concentration was reduced to 2%, and incubation time was shortened to 2 h) to allow partial digestion of the cell wall, which better retained the properties of fluorescence relaxation in Symbiodiniaceae.

### 4.3. Measurement of Flash-Induced Chlorophyll Fluorescence Relaxation Kinetics

Flash-induced chlorophyll fluorescence yield (FF) was measured using a double-modulation fluorimeter (FL-3000, Photon Systems Instruments, Brno, Czech Republic) [46]. A 2 mL sample was placed in a cuvette with 1 cm path length and was continuously stirred with a small magnetic stirrer bar in the dark. Four measuring flashes (8 μs, separated with 200 μs intervals, wavelength of 620 nm) were applied to determine minimum fluorescence in the dark (F_0_), after which a single-turnover (ST) saturating actinic flash (30 μs, wavelength of 639 nm) was produced to induce the formation of Q_A_^−^, which resulted in the rise in fluorescence intensity (denoted as F_m(ST)_). The fluorescence relaxation resulting from the reoxidation of Q_A_^−^ was measured by applying measuring flashes in the time range from 150 μs to 100 s on a logarithmic timescale.

### 4.4. P700⁺ Reduction Kinetics

P700⁺ reduction kinetic traces were measured using Dual-PAM, Heinz-Walz GmbH, Effeltrich, Germany. A 2 mL sample was placed in a cuvette with 1 cm path length between the emitter and detector, which were placed in 180°, and was dark adapted. The sample was then exposed to a strong actinic illumination (2000 µmol photons m^−^² s^−1^) for 1500 ms, forming the photo-oxidized P700⁺, and then finally, 1000 ms of the dark phase, which shows how fast the electrons can re-reduce the P700⁺ formed. This technique, when performed in the presence of DCMU, which blocks the electrons coming from PSII, has been used to examine cyclic electron flow in Symbiodiniaceae recently [15].

### 4.5. Inhibitors

In our study, we used different electron transport inhibitors in order to characterize the various pathways of photosynthetic electron transport in Symbiodiniaceae. The inhibitors were purchased from Sigma-Aldrich, and the following concentrations were used: 2 µM/µg Chl of 3-(3,4-dichlorophenyl)-1,1-dimethylurea (DCMU), 50 µM/µg Chl of 2,6-Dimethoxy-1,4-benzoquinone (DMBQ), 4 mM/µg Chl of methyl viologen (MV), 800 µM/µg Chl hydroxylamine, 5 mM/µg Chl of glycolaldehyde, 80 µM/µg Chl of polymyxin B (PolyB), and 5 µM/µg Chl of antimycin A (AntiA).

### 4.6. Experimental Procedure

For each experiment, 2 mL of the sample was heated to 38 °C for 10 min by the cuvette heating system (Photon Systems Instruments, ThermoRegulator TR2000). The acute heat was followed by microaerobic treatment, and the changes in flash-induced chlorophyll fluorescence relaxation were recorded. Microaerobic condition was created by incubating the sample in the dark with 7 U mL^−1^ glucose oxidase, 60 U mL^−1^ catalase, and 10 mM glucose for 15 min. For P700⁺ reduction experiment, 2 mL of fresh sample was heated to 38 °C for 10 min using the cuvette heating system. These heated samples were further incubated in the dark with DCMU for another 10 min before each measurement. The differences in the fluorescence decay trends were observed in the absence and presence of inhibitors, which were applied after the heat treatment on the same sample in both of the experiments; in the case of flash-induced chlorophyll fluorescence, they were added along with microaerobic treatment.

## Data Availability

Data is contained within the article or Supplementary Material.

## Supplementary Materials

The supporting information can be downloaded at https://www.mdpi.com/article/10.3390/ijms24108712/s1.
