# Supplementary material for: Characterization of the Flash-Induced Fluorescence Wave Phenomenon in the Coral Endosymbiont Algae, Symbiodiniaceae
Source: Int J Mol Sci. 2023 May 13;24(10):8712. doi: 10.3390/ijms24108712 (PMC10217972; doi:10.3390/ijms24108712)
Supplement: Supplementary file 1 [file ijms-24-08712-s001.zip › Aslam2023 IJMS Supplementary Material.pptx]

## Slide 1
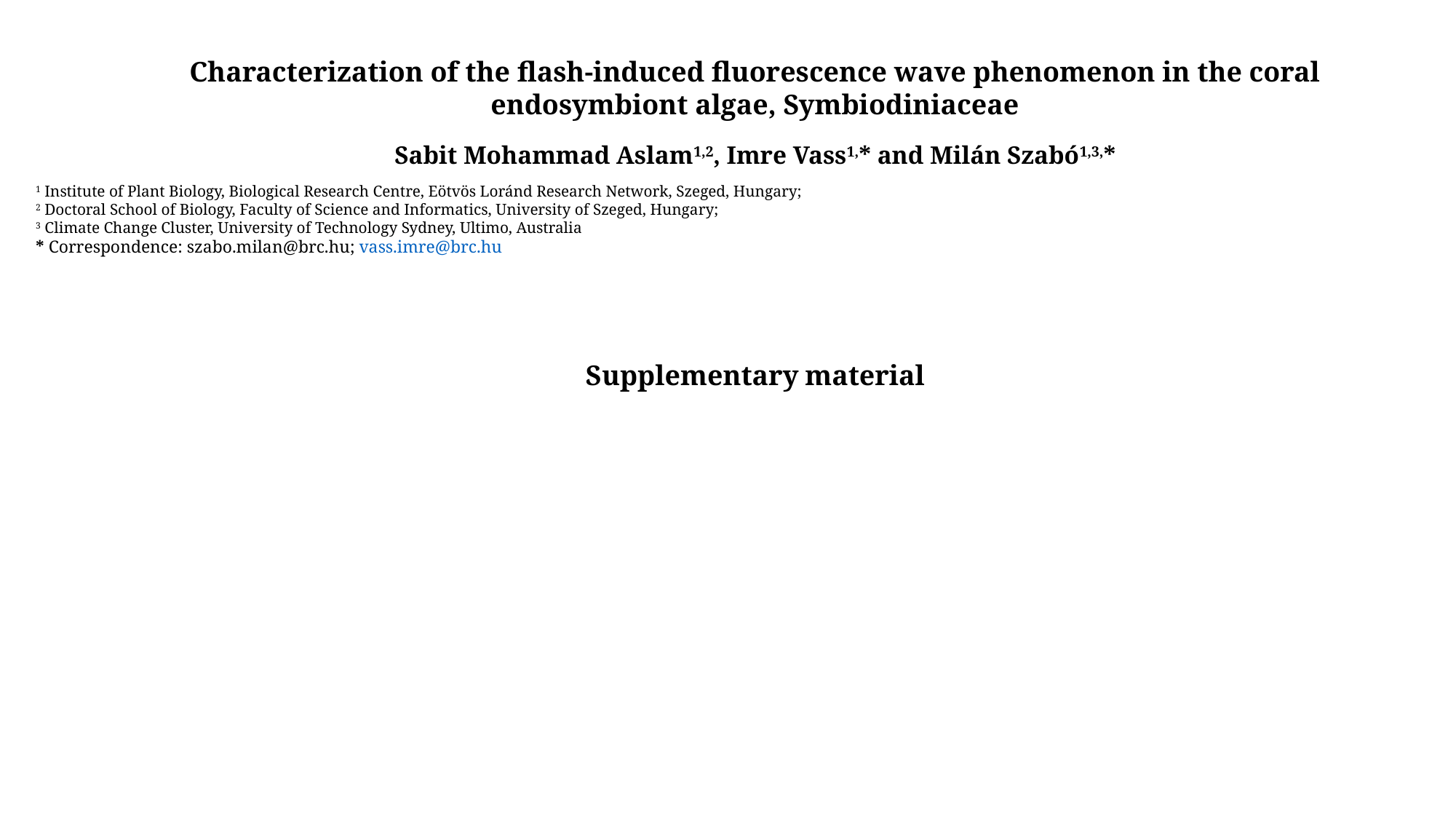

Characterization of the flash-induced fluorescence wave phenomenon in the coral endosymbiont algae, Symbiodiniaceae
Sabit Mohammad Aslam1,2, Imre Vass1,* and Milán Szabó1,3,*
1 Institute of Plant Biology, Biological Research Centre, Eötvös Loránd Research Network, Szeged, Hungary;
2 Doctoral School of Biology, Faculty of Science and Informatics, University of Szeged, Hungary;
3 Climate Change Cluster, University of Technology Sydney, Ultimo, Australia
* Correspondence: szabo.milan@brc.hu; vass.imre@brc.hu
Supplementary material

## Slide 2
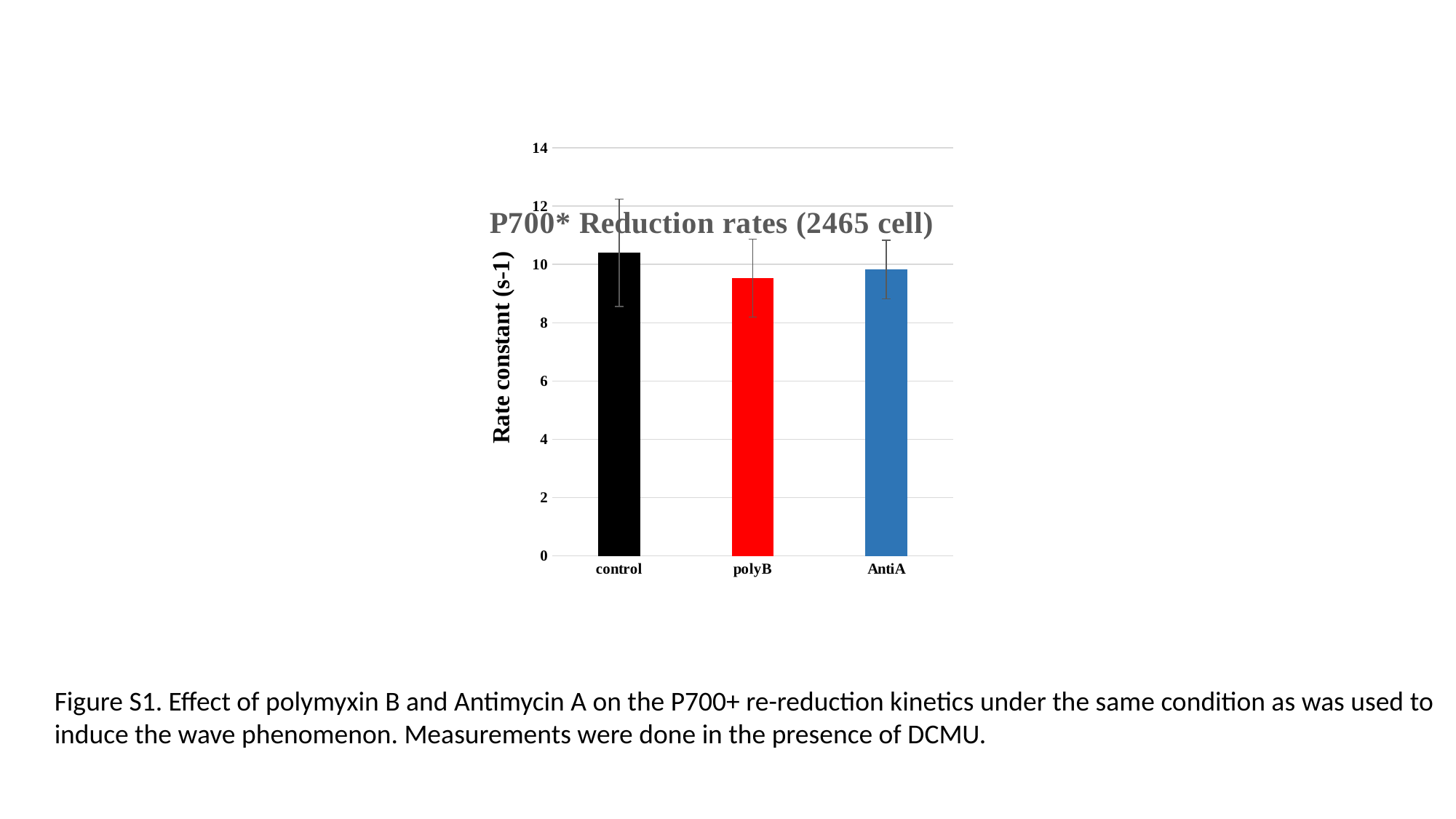

### Chart
| Category | avg |
|---|---|
| control | 10.395577296581335 |
| polyB | 9.53230986310497 |
| AntiA | 9.826709335344901 |
### Chart: P700* Reduction rates (2465 cell)
| Category |
|---|Figure S1. Effect of polymyxin B and Antimycin A on the P700+ re-reduction kinetics under the same condition as was used to induce the wave phenomenon. Measurements were done in the presence of DCMU.

## Slide 3
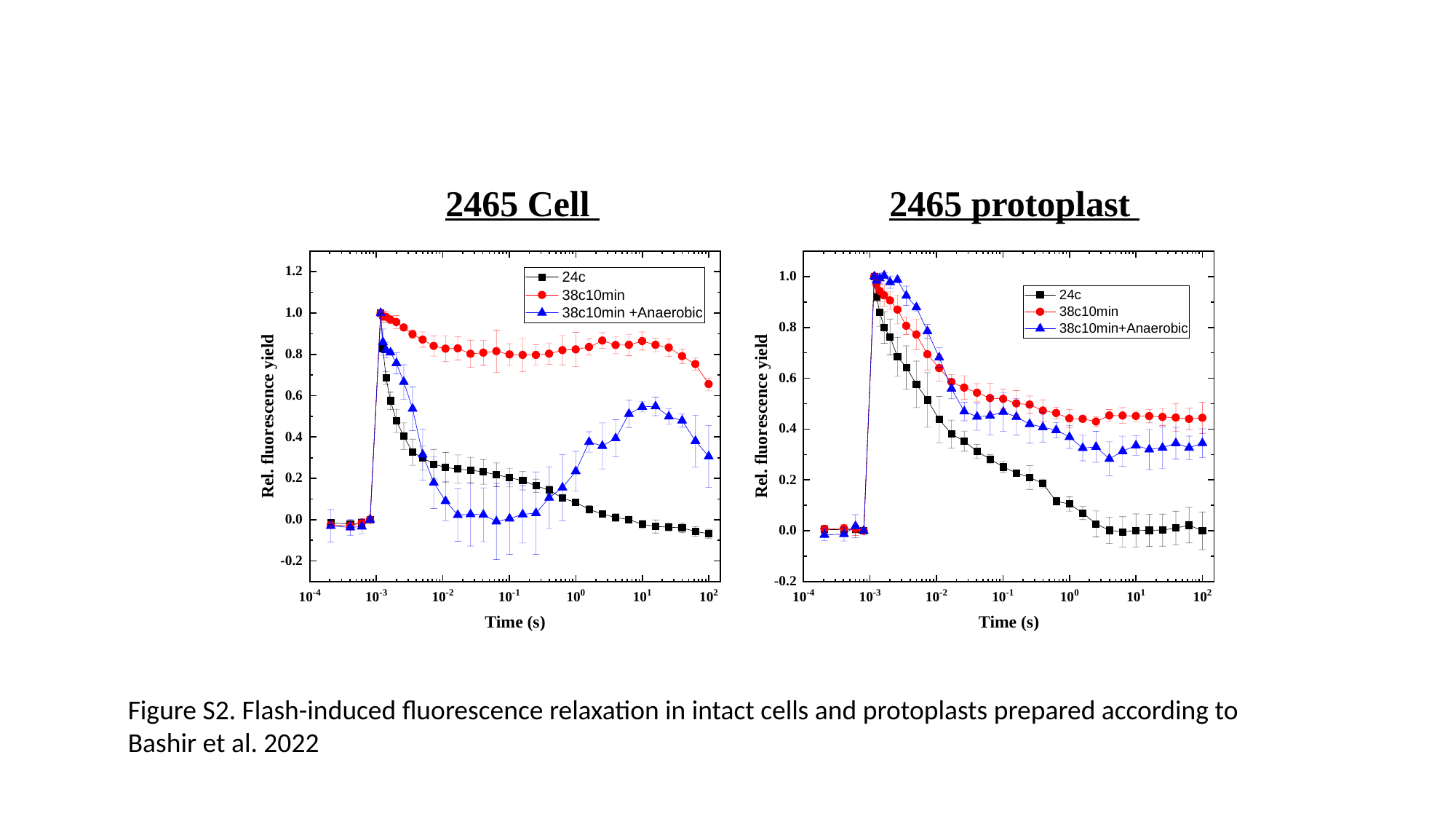

2465 Cell
2465 protoplast
Figure S2. Flash-induced fluorescence relaxation in intact cells and protoplasts prepared according to Bashir et al. 2022

## Slide 4
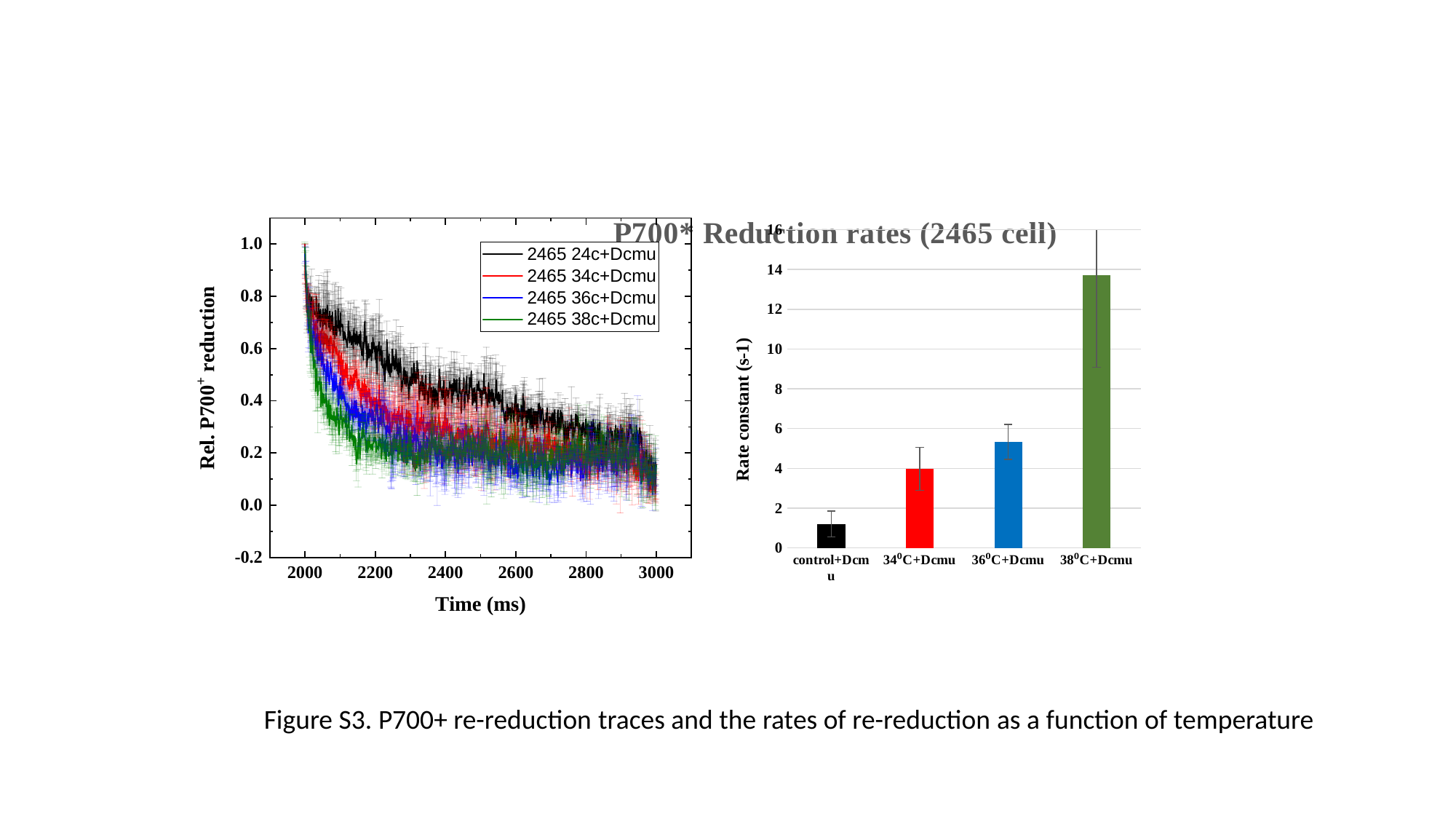

### Chart: P700* Reduction rates (2465 cell)
| Category |
|---|
### Chart
| Category | |
|---|---|
| control+Dcmu | 1.1981097061495956 |
| 34⁰C+Dcmu | 3.9799960585963507 |
| 36⁰C+Dcmu | 5.332074087678532 |
| 38⁰C+Dcmu | 13.69679485378849 |Figure S3. P700+ re-reduction traces and the rates of re-reduction as a function of temperature
